# Notes and News

**Published:** 1902-10-18

**Authors:** 


### NOTES AND NEWS.

On October 21st the Princess Christian will lay the foundation stone of the new Hampstead Hospital.

The new Dundee sanatorium for consumptives has been opened near Auchterhouse.

Major Ross has sent a very interesting preliminary report of his investigations into malaria, at Ismailia, to the Liverpool School of Tropical Medicine. He thinks that the extermination of the malaria mosquito will be a comparatively easy task, to be effected by filling up the small marshes, or deepening them to admit fish. As a temporary expedient the pools are being treated with oil.

At a recent meeting of the Metropolitan Asylums Board an interesting report was presented, dealing with the question of asylum phthisis as it occurs among the imbeciles of whom the Board has charge. The question put to Dr. Shadwell, the author of the report, had been as to the comparative suitability of the managers' imbecile asylum at Darenth and the Brighton Road Schools at Sutton, as places to which cases of tuberculosis might be drafted as they occurred or were discovered in other asylums. In answer Dr. Shadwell pointed out that if tuberculous imbeciles were to be segregated in any one spot, he would recommend the Darenth Asylum in preference to any of the others. But after a careful review of the whole question he did not feel able to advise the committee to adopt the proposed course. The most advanta geous plan was in his opinion to attack the mischief at its source and deal with the conditions within the asylums, which at present conduced to the propagation of tubercle. The measures he suggested were the removal from the general wards of patients suffering from active tubercle, and their treatment in special blocks, the better provision of fresh air, and the elimination of dust. Such measures, he said, could not fail to produce a great diminution of tuberculosis, and without them the establishment of a sanatorium would be of little use. At present he thought the problem was not ripe for such a step as segregating the tuberculous patients in one institution.

King Edward's Hospital Fund has received £2,500 from the executors of the late Mr. E. A. Newman.

A meeting of old St. George's men is to take place on the 16th instant to consider measures to increase the funds of the hospital. The income from subscriptions has been gradually falling off.

A new wing has been added to the Newcastle-upon-Tyne Lying-in Hospital, dedicated to the memory of the late Dr. Nesham, for thirty-three years physician of the hospital.

The Odontological Society of Great Britain is prepared to receive applications for grants in aid of the furtherance of scientific research in connection with dentistry. The address of the society is 20 Hanover Square.

The Paris Assistance Publique has appointed M. Mesureur as its new director. M. Mesureur is prepared to carry out the reforms initiated by the late director, and this will entail the Assistance being placed largely under the control of the Municipal Council, who will advance the necessary funds to carry out the proposed alterations.

The Rugby Rural District Council did not receive much sympathy from the public auditor for their efforts to prevent the spread of small-pox. A payment had been made by them to some "contacts" to induce them to remain at home. The payment did not amount to a large sum, but it was nevertheless disallowed by the auditor.

Dr. Cullingworth will deliver the Bradshaw Lecture before the Royal College of Physicians on November 5. The Cronian Lectures, which will be delivered by Dr. Hale White, were prepared by the late Dr. J. Washbourn, the subject being "National History and Pathology of Pneumonia." The Harveian Oration will be delivered by Dr. D. Ferrier on October 18th.

Amongst the many agencies created to assist the sufferers from the South African War few have carried on their work more quietly and effectively than Georgina Lady Dudley's Convalescent Fund for Officers and Nurses. The help that has been given is immense, and 1,111 cases have been helped. This figure includes many officers, their wives and children and 314 nurses and 28 friends who have been paid for that they might attend sick nurses when necessary. The cost of the sick officers has been very heavy, as many severe and complicated operations have been entailed. Lady Dudley has met with a very generous response to her appeals; but the war is now over, and there are many cases still requiring assistance, without which their chances of recovery are imperilled. Lady Dudley therefore appeals once more for help to complete her good work, which, in all probability, can be accomplished in a few months' time. She certainly should not have to plead in vain.

